# A Population-based survey of the prevalence and types of glaucoma in Nigeria: results from the Nigeria National Blindness and Visual Impairment Survey

**DOI:** 10.1186/s12886-015-0160-6

**Published:** 2015-12-12

**Authors:** Fatima Kyari, Gabriel Entekume, Mansur Rabiu, Paul Spry, Richard Wormald, Winifred Nolan, Gudlavalleti V. S. Murthy, Clare E. Gilbert, On behalf of the Nigeria National Blindness and Visual Impairment Study Group

**Affiliations:** International Centre for Eye Health (ICEH), Clinical Research Department, London School of Hygiene and Tropical Medicine (LSHTM), Keppel Street, London, WC1E 7HT UK; Department of Ophthalmology, College of Health Sciences (CHS), University of Abuja, Abuja, Nigeria; Vision Health Services, Ikeja, Lagos State Nigeria; Prevention of Blindness Union, Riyadh, Saudi Arabia; Bristol Eye Hospital, University Hospitals, Bristol NHS Foundation Trust, Bristol, UK; Moorfields Eye Hospital, London, United Kingdom; Indian Institute of Public Health, Public Health Foundation of India, Hyderabad, Andhra Pradesh India

**Keywords:** Prevalence, Glaucoma, Epidemiology, Nigeria, Open-angle glaucoma, Angle-closure glaucoma

## Abstract

**Background:**

Glaucoma is the leading cause of irreversible blindness worldwide. There tends to be a lower reporting of glaucoma in Africa compared to other blinding conditions in global burden data. Research findings of glaucoma in Nigeria will significantly increase our understanding of glaucoma in Nigeria, in people of the West African diaspora and similar population groups. We determined the prevalence and types of glaucoma in Nigeria from the Nigeria National Blindness and Visual Impairment cross-sectional Survey of adults aged ≥40 years.

**Methods:**

Multistage stratified cluster random sampling with probability-proportional-to-size procedures were used to select a nationally representative sample of 15,027 persons aged ≥40 years. Participants had logMAR visual acuity measurement, FDT visual function testing, autorefraction, A-scan biometry and optic disc assessment. Participants with visual acuity of worse than 6/12 or suspicious optic discs had detailed examination including Goldmann applanation tonometry, gonioscopy and fundus photography. Disc images were graded by Moorfields Eye Hospital Reading Centre. Glaucoma was defined using International Society of Geographical and Epidemiological Ophthalmology criteria; and classified into primary open-angle or primary angle-closure or secondary glaucoma. Diagnosis of glaucoma was based on ISGEO classification. The type of glaucoma was determined by gonioscopy.

**Results:**

A total of 13,591 participants in 305 clusters were examined (response rate 90.4 %). Optic disc grading was available for 25,289 (93 %) eyes of 13,081 (96 %) participants. There were 682 participants with glaucoma; a prevalence of 5.02 % (95 % CI 4.60–5.47). Among those with definite primary glaucoma that had gonioscopy (*n* = 243), open-angle glaucoma was more common (86 %) than angle-closure glaucoma (14 %). 8 % of glaucoma was secondary with the commonest causes being couching (38 %), trauma (21 %) and uveitis (19 %). Only 5.6 % (38/682) of participants with glaucoma knew they had the condition. One in every 5 persons with glaucoma (136;20 %) was blind i.e., visual acuity worse than 3/60.

**Conclusion:**

Nigeria has a high prevalence of glaucoma which is largely open-angle glaucoma. A high proportion of those affected are blind. Secondary glaucoma was mostly as a consequence of procedures for cataract. Public health control strategies and high quality glaucoma care service will be required to reduce morbidity and blindness from glaucoma.

## Background

Glaucoma is the second leading cause of blindness worldwide and the leading cause of irreversible blindness, accounting for 8 % of all blindness, affecting an estimated 3.12 million blind people [1]. A review of relevant population-based surveys of glaucoma, and of blindness and visual impairment in sub-Saharan Africa indicate that glaucoma affects about 4 % of adults aged 40 years and above and accounts for 15 % of blindness [2–4]. Africa is the region with the highest incidence and prevalence of glaucoma, most of which is open-angle glaucoma (OAG) [5, 6], and OAG is more prevalent in the black populations of Africa and Africa-derived populations [7]. Reports also suggest that the most difficult problematic OAG that there is in terms of severity of disease, difficulty in treating it and as a cause of blindness comes from West Africa [2, 3, 8]. Additionally, there tends to be a lower reporting of glaucoma in Africa compared to other blinding conditions in global burden data because surveys in Africa may have had limited diagnostic capacity for glaucoma [9]. The Nigeria national blindness and visual impairment survey (hereafter referred to as the Nigeria Blindness Survey), in which over 13,500 people aged 40 years and above were examined, is a population-based survey that substantially addresses glaucoma prevalence and risk factors. The Nigeria Blindness Survey reported the prevalence of blindness to be 4.2 % (95 % confidence interval 3.8–4.6 %) [10], 16.7 % being due to glaucoma [11]. Glaucoma was the leading cause of irreversible blindness [11] and functional low vision [12].

A standard definition and classification system for glaucoma in prevalence surveys proposed by the International Society of Geographical and Epidemiological Ophthalmology (ISGEO) [13] allows comparison of glaucoma prevalence surveys, further highlighting the variation between populations. Whereas angle-closure glaucoma is more frequent among east Asian populations [6, 14], the black populations of USA [15] the Caribbean [16, 17], and Africa [6, 18–22] have the highest prevalence of open-angle glaucoma with up to 90 % of those affected being unaware that they have the condition [18, 21, 22].

In this study, data from the Nigeria Blindness Survey were analyzed using ISGEO criteria to determine the prevalence and types of glaucoma, to provide data for advocacy, policy and to plan services for glaucoma. However, the ISGEO classification system is not for clinical diagnosis or for assessment for treatment of glaucoma. The percentile values for the vertical cup:disc ratio (VCDR), VCDR asymmetry and intraocular pressure (IOP) to define glaucoma were derived from this study population. Possible risk factors for glaucoma in the population are presented in another paper.

Nigeria is the 7^th^ most-populous country in the world and had a total population of 128 million at the time of the national survey (January 2005 to June 2007). Nigeria has 6 main administrative/geo-political zones (GPZ): north-east (NE), south-east (SE), south-south (SS), north-west (NW), south-west (SW) and north-central (NC). Two-thirds (63 %) of the population live in rural areas. Nigeria has more than 250 ethnic groups, who live in different areas in the country each with their own language/dialects, customs and practices. The largest ethnic groups are the Hausa and Fulani in the north, Ibo in the south-east and Yoruba in the south-west. Despite recent economic development, adult literacy levels remain low (51 %), and 54 % of the population live below the poverty line on less than a dollar a day [23].

There are insufficient population-based glaucoma studies in Africa to represent the entire continent in global glaucoma prevalence estimates [6]. From the few high-quality surveys, it is difficult to extrapolate the findings to wider populations as they were conducted in limited and defined geographical areas of large countries [3]. This is the largest truly population-based study of glaucoma in Africa. Estimating the magnitude of glaucoma in Nigeria is important because it sheds light on inter-ethnic and regional variations of OAG prevalence in the black populations of Africa, Caribbean and USA. It will also provide a baseline for planning delivery of care to glaucoma patients in Nigeria and in countries with similar socio-demographic and ecological characteristics in sub-Saharan Africa.

## Methods

Details of all the methods used in the Nigeria Blindness Survey have been published [24] as well as data on the prevalence [10] and causes of visual impairment, blindness [11] and low vision [12].

### Ethics

Ethical approval was obtained from the Ethics Committee of the London School of Hygiene & Tropical Medicine and the Federal Ministry of Health, Nigeria. Oral informed consent was obtained from community leaders, heads of households and all participants. The study adhered to the tenets of the declaration of Helsinki. Persons with medical or eye conditions needing further assessment and treatment were referred to the nearest healthcare facility. Cataract blind participants were offered surgery after the survey had been completed in each zone.

### Sample size calculation and sampling strategy

The sample size calculation was based on the following: target population (22.6 million); expected prevalence of blindness in persons 40 years and older (5 %); desired precision (0.5 %); design effect due to clustered sampling (2); 95 % confidence level; and 85 % response rate. The sample size was 15,375 after allowing for non-response in 310 clusters of 50 participants each. With assumed glaucoma prevalence of 5 % [3], this sample size would also give a precise estimate of the prevalence of glaucoma and allow risk factors for OAG to be analysed.

Multi-stage sampling using probability in proportion to size was used to select a nationally representative sample. In each cluster the center of the village/ward was identified and the direction of enumeration determined by spinning a bottle. Individuals aged 40 years and above who had lived in the household for at least the preceding 3 months were enumerated until 50 individuals had been identified. Examination took place over two days in a temporary clinic set up in the community. Those unable to leave their homes (e.g., due to disability) were examined at home.

### Clinical teams and quality control

Data were collected by two clinical teams each comprising of two ophthalmologists, one optometrist, two ophthalmic nurses, four enumerators and one interviewer. Quality assurance included field supervision by the team leader, daily review of data collection forms, frequent visits by the Project Manager (MR) and Project Epidemiologist (GVSM), inter-observer agreement studies, retraining of all team members before visiting each zone, and double data entry by two trained data entry personnel. Three of the four ophthalmologists and both optometrists comprising the clinical teams remained unchanged but different nurses were recruited for each zone in order to address language and cultural variations. A detailed protocol of all the methods was used in training and for reference.

### Data collection and clinical assessment

**Fig. 1:**
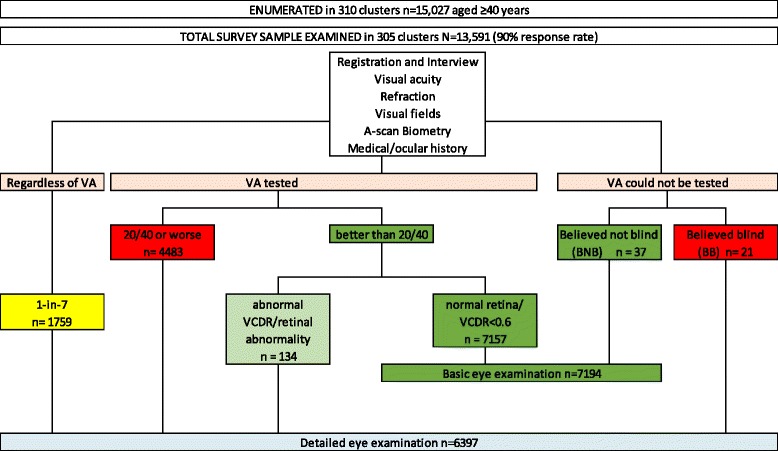
Examination flow chart for study participants in the Nigeria national survey of blindness and visual impairment. VA = visual acuity; BNB = believed not blind; BB = believed blind, VCDR = vertical cup:disc ratio. Basic eye examination: (n=7194)- Pen-torch anterior segment examination, non-dilated direct ophthalmoscopy. Detailed eye examination: (n=6397) - Slit-lamp examination, WHO lens grading, Van Herick's anterior chamber angle depth estimation, applanation tonometry, gonioscopy if indicated, dilated ophthalmoscopy, digital retinal photography

Clinical assessment in relation to glaucoma is described below. The examination flow is shown in Fig. 1.

#### All participants

All participants had their personal and socio-demographic data recorded including their self-reported ethnic group as well as medical and ocular history, including a history of glaucoma. Height, weight and blood pressure (Omron) were measured. Presenting and best-corrected distant visual acuities (VA) were measured by an ophthalmic nurse with a reduced logMAR E-chart [25]. All participants also had automated refraction, frequency doubling technology (FDT) visual field testing (see below), A-scan biometry by the optometrist and a basic eye examination by the first ophthalmologist.

#### Detailed examination

The following participants underwent detailed eye examination by the second ophthalmologist [10, 24]: those with a presenting VA <6/12 in one or both eyes; VCDR ≥0.6 in one or both eyes or VCDR asymmetry of ≥0.2, or any retinal abnormality seen on non-dilated direct ophthalmoscopy. In addition, 1-in-7 participants also had the detailed examination regardless of their VA, with random blood glucose testing, to provide a ‘normative’ database. Detailed eye examination included slit-lamp examination (Zeiss SL 115 Classic Slit Lamp, Carl Zeiss Meditec AG Jena Germany), Van Herick’s (VH) anterior chamber (AC) angle depth estimation [26], assessment for relative afferent pupil defect (RAPD), applanation tonometry (Goldmann), lens opacity grading using the World Health Organization (WHO) classification, fundus and optic disc examination with 60D aspheric condensing lens (Volk) and binocular indirect ophthalmoscopy (BIO; Keeler all-pupil) with a 20D lens, and digital fundus imaging with Zeiss Visucam Lite Desk Top Fundus Camera (Carl Zeiss Meditec AG Jena Germany) focused on mid-point between the optic nerve head and the macular region through a dilated pupil. All images were graded independently at the Moorfields Eye Hospital Reading Centre (MEHRC). Gonioscopy (Volk’s 1-mirror non-flanged lens) was performed if the IOP was ≥20 mmHg, or VCDR ≥0.6, or VCDR asymmetry ≥0.2, or VH grades 0, 1, 2. Central corneal thickness was not assessed.

### Visual field testing

Visual field testing was performed with a Humphrey FDT visual field analyzer (Carl Zeiss Meditec AG Jena Germany). The FDT perimeter is a robust, portable, self-contained unit that weighs less than 10 kg and has a self-calibration procedure. It is generally inexpensive, easy to understand and quick to perform the test [27]: it takes about 45 s to complete a normal screening test and about 45 s for a normal threshold test. These features informed the choice of the FDT perimeter and were advantages considering the logistics of a large population-based survey of this kind where examinations were carried out in temporary examination centers set up in the community. FDT utilizes a vertical sine wave grating of low spatial frequency (0.25 c/deg) with counterphase flickering at a high temporal frequency (25 Hz) [28]. All participants were screened using the suprathreshold (C20-5 or C20-1) screening mode after explaining the test and running a demonstration. Each eye was tested separately without correction. The reliability indices considered were fixation error and false positive. The screening test was stopped and restarted or repeated if considered unreliable i.e., there were two or three false positives and/or two or more fixation errors. Clinically abnormal tests were not repeated. A threshold test was done if there were ≥3 field defects at *p* <1 % or ≥2 field defects at *p* <0.5 %. If a participant could not be tested or could not see the FDT flickering black and white patterns, s/he was classified as having no FDT test and a reason was given e.g., cataract; or did not understand the test. Print-outs of all FDT tests were obtained immediately and data were extracted and entered into a database. Perimetry results were interpreted using a detailed specific algorithm (devised and adapted [27] by PGS and FK) to identify abnormal visual fields and to classify defects as glaucomatous or non-glaucomatous. The criteria used are outlined in Table 1. The FDT result was interpreted by a 1^st^ reader (PM) and validated by a 2nd (FK); any discrepancy was adjudicated (PGS). Screening reliability was defined as ≤1 fixation error and/or ≤1 false positive (i.e., <33 % failed reliability indices) and threshold reliability was defined as ≤2 fixation errors, ≤2 false positives (i.e., ≤33 % errors on reliability indices). Tests were also considered unreliable if there were brow/lid positions showing as uniformly dense artefact along the upper or lower edges of the FDT result chart. Unreliable results were not included.

**Table 1:** Definition of glaucomatous visual field defects for level 1 evidence of glaucoma

FDT test defects	Visual fields				
	Normal	Definitely glaucoma	Probably glaucoma	Possibly glaucoma	Unlikely glaucoma^a^
*P* <5 %	2 or less non-adjacent	4	3	2 adjacent	
*P* <2 %	1	3	2	1	
*P* <1 %	0	2	1 non-edge		
*P* <0.5 %	0	1	1 non-edge		
Comments		At any location in any hemi-field	At one hemi-field	At one hemi-field	If TDP plot is better than PDP plot
Participants with glaucoma	Total				
Number of participants^b^	268 (100 %)	252 (94 %)	6 (2.2 %)	9 (3.4 %)	1 (0.4 %)
Number of eyes	310 (100 %)	283 (91.3 %)	9 (2.9 %)	13 (4.2 %)	5 (1.6 %)

*TDP* total deviation probability, *PDP* pattern deviation probability

^a^Other evidence of glaucoma noted in those classified as glaucoma

^b^In participants with bilateral glaucoma, the eye with the highest level of evidence is used to classify that person

Threshold test results were used to diagnose glaucoma if available, otherwise screening results were used. Grading used defects on the Pattern Deviation Probability (PDP) plot compared with the Total Deviation Probability (TDP) plot. Screening tests were considered normal if reliable without defects, or there were ≤2 defects at *p* <1 %; or ≤1 defect at *p* <0.5 %. Threshold tests were normal if there were no defects at *p* <0.5 % and *p* <1 %, or ≤1 defect at *p* <2 %, or ≤2 non-adjacent defects at *p* <0.5 %. Factors considered in categorizing defects as definitely, probably or possibly glaucomatous were position, depth and size, clustering (i.e., adjacent or not) and position; and repeatability (i.e., defect in same location on PDP and TDP plots). We could determine repeatability in participants that had both screening and threshold tests. Defects were not likely glaucomatous if 1) there was a highly shaded TDP with normal PDP plot – this diffuse loss could be due to cataract, for example; 2) TDP was normal or better than the PDP plot or 3) there were vertical meridian defects. However, for diffuse defects, other compelling evidence for glaucoma classification were used (see later).

### Van Herick’s anterior chamber angle estimation and gonioscopy

The VH AC angle estimation was performed at the slit-lamp. The relationship between the corneal slit image and AC depth was graded 0 to 4 [26]. Grades 0, 1 and 2 were grouped as angle closure or likely to close angles; and grades 3 and 4 as open angles. The iridocorneal angle was assessed by gonioscopy without corneal compression and graded as either open angle or closed angle. The anterior chamber angle was classified as open when Schwalbe’s line could be seen; and as closed when it could not be seen. In eyes with glaucoma, the correlation between VH grades and gonioscopy was assessed with the kappa statistic.

### IOP measurement

Intraocular pressures were measured by Goldmann applanation tonometry using standard methods and recorded to the nearest 1 mmHg. Tonometers were checked for calibration daily according to the manufacturer’s recommendation. Eyes with significant corneal surface pathology, phthisis or participants unable to fixate were excluded.

### Optic disc assessment

Cup-disc ratios were assessed clinically by direct ophthalmoscopy for all participants during the basic examination, and after pupil dilation in those having the detailed examination using slit lamp biomicroscopy with a 60D lens. Clinical grading was used in analysis for participants that did not have photo VCDR grading. Methods for clinical VCDR grading by the ophthalmologists were standardized during training using standard sets of optic disc photographs and comparing the clinical grading with the VCDR measured on the retinal photo of the participant being observed.

Digital fundus images were graded independently by MEHRC using their standard protocol. Images were viewed "full screen" on either a 24-in. Eizo S2433W monitor or on a 24-in. widescreen Dell 2407WFP LCD monitor. The former was calibrated using a Datacolor Spyder2 calibrator and the latter was calibrated using a GretagMacbeth Eye-One Display2 calibrator. After determining image quality and clarity, the scleral rim was identified and the boundaries of the disc and cup identified using monocular clues such as vascular change in direction. Disc pallor gave few clues and was not used. The VCDR was then quantified. One successful measurement was performed per eye, along the vertical meridian, in Adobe Photoshop (version 7) using the measurement tool, resulting in a cup and a disc diameter value in proprietary units, the division of the two values producing the VCDR which was recorded to the nearest 0.05. Primary grading was performed by the 1^st^ reader (FS) and inconclusive cases, e.g., tilted discs, blurred images, generalized disc pallor, were adjudicated by a 2^nd^ reader (TP) immediately. If a VCDR measurement could not be obtained, this was stated.

Inter-observer agreement for clinical VCDR measurement between ophthalmologists was assessed with the kappa statistic; each participant had two observations with the second examiner blinded to the result obtained by the first examiner. Inter-observer agreement for VCDR grading on photos was also assessed. The Bland-Altman method was applied to assess agreement between the two methods of measurement i.e., by biomicroscope funduscopy (clinical VCDR) and digital image analysis (image VCDR).

### Glaucoma diagnostic algorithm

**Fig. 2:**
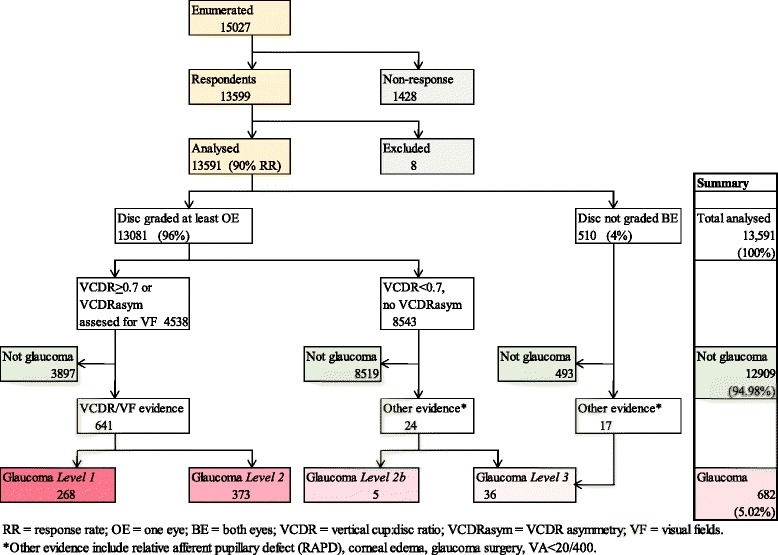
Glaucoma diagnostic algorithm and number of glaucoma participants in each category

Glaucoma was classified according to the ISGEO criteria, using percentile distributions of VCDR, VCDR asymmetry and IOP in *normal* Nigerians, derived from the normative dataset (*n* = 1759) of this study population [29] (Table 2). The diagnosis of glaucoma started with VCDR findings (Fig. 2). Category 1 required structural and functional evidence i.e., 97.5^th^ percentile of the VCDR (≥0.7) or VCDR asymmetry (≥0.1) in our normal population and visual field loss typical of glaucoma. Category 2 required advanced structural damage i.e., 99.5^th^ percentile VCDR (≥0.75) or VCDR asymmetry (≥0.2) in the absence of visual field evidence i.e., when a useful visual field result was not possible or available. Category 3 applied when the optic disc was not seen and visual field testing was not possible, and used: a) blindness (VA <3/60) with the 99.5^th^ percentile IOP (≥28 mmHg), or b) diagnosed with/being treated for glaucoma. An additional level of evidence (level 2b) was added where the optic disc was visualized but the VCDR was <99.5^th^ percentile and visual fields were not available or if visual fields were interpreted as “unlikely glaucoma” but there were other compelling evidence such as RAPD, high IOP and/or corneal edema. Other glaucomatous optic nerve head features such as localized narrowing of the rim, optic disc hemorrhages, and retinal nerve fiber layer defects are not included in the ISGEO classification, and so individuals with these signs only (i.e., no visual field defects; IOP within the normal range for the study population) would not have been classified as having glaucoma. These cases were adjudicated by glaucoma specialists (RW and WN). A person was said to have glaucoma if there was glaucoma in one or both eyes.

**Table 2:** International Society of Geographical and Epidemiological Ophthalmology (ISGEO) definitions for glaucoma used in analysis (Adapted From Foster, 2002) [13]

	VCDR or VCDR asymmetry					
Level of evidence	Image reading analysis	Clinical records analysis	Visual fields	Intraocular pressure	Visual acuity	Medical history
						Other features
Category 1	≥97.5^th^ percentile:	≥97.5^th^ percentile:	Typical defect			
VCDR	0.7	0.6				
VCDR asymmetry	0.1	0.2				
Category 2	≥99.5^th^ percentile:	≥99.5^th^ percentile:	Not available			
VCDR	0.75	0.7				
VCDR asymmetry	0.2	0.3				
Category 2b	≤97.5^th^ percentile: 0.7	≤97.5^th^ percentile: 0.6	±Typical defect	≥99.5^th^ percentile: 28 mmHg		RAPD, Corneal edema
Category 3	Not available		Not available	≥99.5^th^ percentile: 28 mmHg	<20/400	Surgery for glaucoma

*VCDR* vertical cup:disc ratio, *RAPD* relative afferent pupillary defect

### Type of glaucoma

Glaucoma was classified as primary and secondary glaucoma. Primary glaucoma was classified as primary open-angle glaucoma (POAG) or primary angle-closure glaucoma (PACG) according to angle morphology viewed by gonioscopy. Glaucoma was classified as secondary where there was an underlying cause such as AC angle neovascularization, exfoliation, pigment dispersion, trauma, surgical procedure, couching or uveitis. The type was unclassified in eyes that did not have gonioscopy.

### Data analysis and statistical methods

Visual acuities were categorized using the WHO classification of blindness and visual impairment with addition of a category for mild visual impairment (worse than 6/12 but up to 6/18). The classification uses presenting VA in the better seeing eye. Age was categorized in 10-year groups. Any ability to read and write was classified as literate. Ethnic groups represented by ≥200 participants were analyzed separately (i.e., Hausa, Yoruba, Igbo, Fulani, Kanuri, Nupe, Ijaw, Ibibio, Tiv and Urhobo). Ethnic groups with <200 participants were grouped as “Others” and analyzed collectively. Settlements with a population of ≤20,000 were classified as rural.

The percentile VCDR values used for classification of glaucoma in this study were derived from the photo VCDR grades of the ‘normative’ data. The percentile values for the distribution of the clinical VCDR records are included in Table 2 for comparison.

Statistical analysis was performed using Stata (Stata/IC 13.0; Stata Corp, College Station, TX). A descriptive analysis of the study population was undertaken. Univariate analysis was performed to describe socio-demographic characteristics (age, gender, ethnic group, literacy and rural/urban place of residence). The age/sex-specific prevalence of glaucoma with 95 % confidence intervals (CI) was calculated taking account of additional variation introduced by the stratified cluster sampling design. Missing values were indicated and excluded in the analysis.

## Results

A total of 15,027 adults aged ≥40 years were enumerated in 310 clusters, 13,591 (90 %) of whom were examined in 305 clusters. 6,397 participants had detailed eye examination, 3814 (59.6 %) of whom had images for VCDR assessment in both eyes and 817 (12.8 %) in one eye. Where there was no disc image (2624 eyes of 1329 [20.8 %] participants), clinical VCDR grade was used (Table 3). Reasons why there were no disc images are stated in Table 4. Photos were ungradable if no optic disc features could be assessed due to blur or wrong field definition. Clinical VCDR grades were also used in participants undergoing the basic eye examination only. In the whole study sample, a total of 25,289 (93 %) eyes of 13,081 (96 %) participants had photographic or clinical VCDR grades; 510 (4 %) participants did not have VCDR graded in both eyes (Fig. 2).

**Table 3:** Summary of completeness of data for participants undergoing full examination (*N* = 6397)

	Eye level data						Person level data			
	Right eye		Left eye		All eyes		One/both eyes		Both eyes	
	*N*	%	*N*	%	*N*	%	*N*	%	*N*	%
Total	6397		6397		12,794		6397		6397	
Examination										
Van Herick’s	5830	91.1	5821	91.0	11,651	91.1	5967	93.3	5684	88.9
Intra-ocular pressure	5496	85.9	5478	85.6	10,974	85.8	5638	88.1	5336	83.4
Disc grading										
Photo	4203	65.7	4242	66.3	8445	66.0	4631	72.4	3814	59.6
Clinical	1320	20.6	1304	20.4	2624	20.5	1329	20.8	993	15.5
None	874	13.7	851	13.3	1725	13.5	–	–	437	6.8

**Table 4:** Reasons why there was no photo disc grading in 4349 (34 %) eyes among those who had full examination (*n* = 12,794 eyes)

Reason	Right eye	Left eye	All eyes	%
Eye disease				
Cataract	552	469	1021	24 %
Corneal opacity	304	295	599	14 %
Other ocular pathology	166	192	358	8 %
			1978	46 %
Participants factors				
Uncooperative	27	31	58	1 %
Other e.g., home visit	31	27	58	1 %
			116	2 %
Technical reasons				
Faulty camera	471	472	943	22 %
No electricity	144	144	288	7 %
			1231	28 %
Other				
No reason stated	271	312	583	14 %
Ungradable photos^a^	228	213	441	10 %
			1024	24 %
Total	2194	2155	4349	100 %

^a^Photos were taken but VCDR could not be assessed because of blurred image due to media opacity or poor positioning of the participant

The kappa for inter-observer agreement on ophthalmologists’ clinical measurement of VCDR within 0.1 was κ = 0.86 (almost perfect agreement) and classifying ≥0.6 or <0.6 was κ = 0.47 (moderate agreement). Overall, the inter-observer agreement between graders for the image VCDR grading at MEHRC was 99.7 %. The Bland-Altman limits of agreement between the clinical and the image VCDR measurements for 95 % of eyes were lower limit of −0.2 to upper limit of 0.3; and 93 % eyes had a difference of ≤0.25 between the two methods of VCDR measurement. In participants undergoing detailed eye examination (*n* = 6397), 93 % and 88 % had VH AC depth estimation and IOP measurement in at least one eye, respectively (Table 3). With 94 % agreement, the kappa for correlation of gonioscopy (closed/open) Vs VH AC (grades 0–2/3–4) in 397 eyes with glaucoma was κ = 0.70 (substantial agreement).

As shown in Fig. 2, 770 participants had VCDR ≥ 0.7 in one or both eyes and a further 3768 had VCDR asymmetry ≥ 0.1, thus a total of 4995 eyes in 4538 participants required visual field analysis (for level 1 evidence) which were available for 3016 (60.4 %) eyes of 2725 (60.1 %) persons. Glaucoma was diagnosed in 63 % (485/770) participants with VCDR ≥0.7/0.75, and in 4.1 % (156/3768) participants with VCDR asymmetry. Other participants were assessed for level 2b and level 3 evidence. The diagnosis of glaucoma was made in a total of 950 eyes of 682 participants - by photo VCDR in 352 (51.6 %), clinical VCDR in 294 (43.1 %) and the disc was not seen in 36 (5.3 %). Thus, glaucoma diagnosis was made by level 1 evidence in 268 (39.3 %), level 2 evidence in 373 (54.7 %), level 2b in 5 (0.7 %) and level 3 in 36 (5.3 %) participants (Table 5).

**Table 5:** Classification of participants with glaucoma by levels of evidence (as described in Table 2)

	Participants with glaucoma	
Level of evidence	Number of participants	Number of eyes
Category 1		
VCDR	155 (22.7 %)	197 (20.8 %)
VCDR asymmetry	113 (16.6 %)	113 (11.9 %)
Total	268 (39.3 %)	310 (32.7 %)
Category 2		
VCDR	330 (48.4 %)	511 (53.8 %)
VCDR asymmetry	43 (6.3 %)	43 (4.5 %)
Total	373 (54.7 %)	554 (58.3 %)
Category 2b	5 (0.7 %)	10 (1.0 %)
Category 3	36 (5.3 %)	76 (8.0 %)
Total glaucoma	682 (100 %)	950 (100.0 %)

*VCDR* vertical cup:disc ratio

### Prevalence and types of glaucoma

The prevalence of glaucoma of all types was 5.02 % (95 % CI 4.60–5.47 %). The prevalence increased with increasing age and was higher in males, those who were not literate and the Igbo ethnic group (Table 6). These differences were statistically significant. The age-specific prevalence and the magnitude of glaucoma in Nigeria derived by direct standardization with the 2012 Nigeria population are shown in Table 7. There are estimated to be 1.2 million Nigerians aged ≥40 years with glaucoma.

**Table 6:** Socio-demographic characteristics of participants with glaucoma in the study population

		Total	Participants with glaucoma		
		*N*	*N*	%	95 % CI
Total		13,591 (100 %)	682	5.02	4.60–5.47
Socio-demographic factors					
Age group (years)	40–49	4889	93	1.90	1.55–2.33
	50–59	3577	130	3.63	3.03–4.36
	60–69	2773	178	6.42	5.50–7.48
	70–79	1653	178	10.77	9.24–12.52
	80+	699	103	14.74	12.31–17.54
					*p <0.001*
Gender	Female	7345	328	4.47	3.98–5.00
	Male	6246	354	5.67	5.05–5.47
					*p = 0.002*
Ethnic group^a^	Hausa	3375	130	3.85	3.00–4.93
	Yoruba	2669	156	5.84	4.94–6.90
	Igbo	1918	149	7.77	6.57–9.16
	Fulani	840	30	3.57	2.53–5.01
	Kanuri	353	18	5.10	3.40–7.58
	Tiv	342	11	3.22	2.29–4.51
	Ijaw	251	15	5.98	4.46–7.96
	Urhobo	245	7	2.86	1.50–5.37
	Ibibio	212	12	5.66	2.35–13.03
	Nupe	211	11	5.21	3.41–7.88
	Others	3117	139	4.46	3.72–5.33
					*p <0.001*
Literacy	Literate	5925	248	4.19	3.60–4.86
	Illiterate	7666	434	5.66	5.14-6.23
					*p = 0.001*
Place of residence	Rural	10,540	520	4.93	4.46–5.46
	Urban	3051	162	5.31	4.47–6.30
					*p = 0.473*
Visual status	Not blind	13,022	546	4.19	3.83–4.59
	Blind	569	136	23.90	20.24–27.99
					*p <0.001*

CI = confidence interval

^a^58 missing values excluded

**Table 7:** Age-standardized glaucoma prevalence rates

	Study sample		Prevalence of glaucoma				Magnitude of glaucoma
			Crude rate		Age-adjusted rate^a^		Estimated numbers
	*N*	%	*N*	%	%	95 % CI	
Age group (years)							
40–49	4889	35.97	93	1.90	1.51	1.96–2.94	166,308
50–59	3577	26.32	130	3.63	3.69	2.98–4.29	232,792
60–69	2773	20.40	178	6.42	8.85	3.99–5.43	318,689
70–79	1653	12.16	178	10.77	16.85	5.91–8.00	321,820
80+	699	5.14	103	14.74	12.32	14.72–20.98	181,807
Total	13,591	100	682	5.02	5.02	4.60–5.47	1,221,416

*CI* confidence interval

^a^Standardized with the 2012 Nigeria population

Among the 243 participants with primary glaucoma classified according to pathophysiology based on AC angle morphology by gonioscopy, 208 (86 %) were classified as POAG and 35 (14 %) as PACG (Table 8). PACG was more common in women but the difference was not statistically significant (*p* = 0.08). There were no differences in age, ethnic distribution or rural/urban place of residence between the two groups. Additionally, there was no statistically significant difference in awareness of having glaucoma (*p* = 0.55): 1 in 8 of those with POAG and 1 in 12 of those with PACG knew they had the disease. IOPs were higher in PACG than POAG: the mean IOP was 34 mmHg, standard deviation (SD) 13 in PACG and 27 mmHg, SD 11 in POAG (*p* <0.001).

**Table 8:** Proportion of the different types of glaucoma in the Nigeria National Survey of Blindness and Visual Impairment

	Proportion of glaucoma	
Glaucoma type	N	%
All glaucoma^a^	682	100.0
POAG	208	30.5
PACG	35	5.1
Secondary glaucoma	53	7.8
Unclassified^b^	386	56.6

*POAG* primary open angle glaucoma, *PACG* primary angle-closure glaucoma

^a^All glaucoma prevalence is 5.02 % (95 % CI 4.60–5.47 %)

^b^No data on gonioscopy, thus not classified by anterior chamber angle morphology

### Other findings

Only 5.6 % (38/682) of all participants with glaucoma knew they had the condition. The commonest causes of secondary glaucoma (*n* = 53 participants) were couching (an ancient, traditional non-medical manipulation of the crystalline lens; 38 %), trauma (21 %), uveitis (19 %) and following intracapsular cataract surgery (17 %). Over a third of the eyes with glaucoma (365; 38 %) had a presenting VA worse than 3/60; and 1 in every 5 persons with glaucoma (136; 20 %) was blind (VA worse than 3/60 in the better eye). In 68 % of the 136 blind with glaucoma, the main cause of blindness was attributable to glaucoma. Among the 40–49 year-group with glaucoma, 13 % were blind, and this age-specific proportion of blindness among glaucoma participants increased with age to 30 % in the 80+ years age-group.

**Fig. 3:**
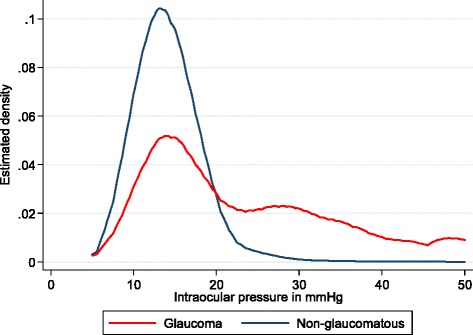
Distribution of IOP in glaucoma and non-glaucomatous eyes

Figure 3 shows the distribution of IOP in all eyes: the mean IOP was 14 mmHg, SD 4 in the non-glaucomatous eyes compared to 23 mmHg, SD 12 in the glaucoma eyes. The difference in the mean values was statistically significant (*p* <0.001). The modal IOP was 12 mmHg in both groups. There were three different peaks at 12, 28 and 50 mmHg in the IOP distribution of the eyes with glaucoma. About half (56 %) of the eyes with glaucoma had IOP ≤22 mmHg. Conversely, 4 % had IOP >21 mmHg but did not have glaucoma.

## Discussion

The Nigeria Blindness Survey was the largest, national population-based survey of eye disease in an ethnically diverse, indigenous black African population, giving precise estimates of the prevalence of glaucoma. The sample was nationally representative by age, gender, ethnicity, rural/urban residence and socioeconomic status [24], with a high response rate and the results are generalizable to the whole country and also to people of the West African diaspora around the world whose predecessors were victims of the slave trade e.g., African Caribbean and African American people. Though now genetically mixed to varying extent, our study population is likely to have the same genetic determinants of the glaucoma seen in those populations. Additional strengths are the standardized protocol, the same clinicians and equipment were used throughout the study, and photographic VCDR grading was performed by the MEHRC, an independent, internationally recognized reading center. Furthermore, the centile values for VCDR and IOP distribution in the population used to define glaucoma were derived from the same study population.

The survey indicates that 1.1 to 1.4 million adults in Nigeria have glaucoma, most of whom are not aware that they have the disease. One in every 20 Nigerians aged 40 years and above has glaucoma, and one in five being blind. There are approximately 8500 people aged 40 years and above with glaucoma per million population. The high prevalence and high rate of blindness confirm glaucoma to be of public health importance and should become a priority among healthcare planners and policy makers, emphasizing the need for glaucoma care pathways for early detection and treatment to prevent blindness. In Nigeria, 8 % of glaucoma was secondary, with over half of these following procedures for cataract, particularly couching, which is still widely practiced in Nigeria despite very poor visual outcomes [30]. This underscores the need for high quality, affordable and accessible cataract surgical services. The findings have public health implications for other countries in sub-Saharan Africa which share similar socio-demographic characteristics.

The prevalence of glaucoma in Nigeria is similar to that in Temba, South Africa [20], slightly higher than in South African Zulus [19] and in Kongwa, Tanzania [18] but lower than in Tema, Ghana [21] and Akinyele, SW Nigeria [22]. Although these surveys were undertaken in localized populations, there seems to be an emerging pattern with the prevalence being higher in West Africa than in South Africa which in turn is higher than in East Africa. The Ghana study [21] had a high proportion of Level 1 diagnosis (87.2 %) compared with our study (39.3 %) as in Nigeria there were high rates of cataract and other pathology which precluded visual field assessment. As Level 2 requires evidence of more advanced structural damage our estimates for Nigeria are, therefore, minimum estimates.

The prevalence of glaucoma in Nigeria is lower than that of POAG reported from Barbados (6.7 %, 95 % CI 6.3–7.8) [17], being similar to black populations in the United States of America (USA) [15] but slightly higher than in Asian populations [31–38] and much higher than white populations in the USA [15, 39], Australia [40] and Europe [41–44]. The prevalence of glaucoma in Nigeria is also higher than in Brazil [45], Iran [46], indigenous populations in Australia [47] and Qatar [48]. Regional/racial variations in prevalence have been attributed to genetic and possible environmental differences [49, 50]. Susceptibility gene loci significantly associated with POAG and genes involved in IOP regulation have been studied in some African populations [51, 52]. In Nigeria, the Igbo, a rather homogenous ethnic group, had the highest prevalence of glaucoma which may also reflect genetic susceptibility.

The relatively high age-specific prevalence of glaucoma in 40–49 year olds in Nigeria and the high proportion of glaucoma blindness suggest severity at an earlier age [15, 17, 21, 22] and more aggressive course [53] in Blacks than in Caucasians [41, 42, 44] and some Asian populations [33, 34] over and above lack of diagnosis and treatment since the high proportions of undiagnosed glaucoma are relatively similar (Table 9). However, this could also be the natural history signifying poor access to treatment. Additionally, because of the earlier age of onset and longer years with untreated glaucoma, the risk of going blind would be much greater. The racial/regional disparity in disease severity may be attributed to additional factors such as inflammation [54, 55] and the different peaks of IOP in eyes with glaucoma may indicate genetic susceptibility at varying levels of IOP. However, these interpretations are speculative and warrant further research.

**Table 9:** Prevalence of Glaucoma in some population-based studies for age ≥40 years

Study population	Examined (response rate %)	Prevalence of glaucoma			Undiagnosed glaucoma (%)	Proportion blind (%)	Reference
		n	All glaucoma % (95 % CI)	40–49 years age-specific			
Nigeria, National	13,951 (90)	682	5.0 (4.6–5.5)	1.9 (1.6–2.3)	94	20	This study
Africa							
Kongwa, Tanzania	3247 (89)	135	4.2 (3.5–4.9)	1.7 (1.1–2.5)	98	14	[18]
Hlabisa, South Africa	1005 (90)	41	4.5 (3.2–6.1)	1.2 (0.2–3.4)	90		[19]
Temba, South Africa	839 (75)	55	5.3 (3.9–7.1)	1.1^b^	87		[20]
Tema, Ghana	5603 (82)	32	6.5 (5.8–7.1)	3.2 (2.7–4.1)	97	3	[21]
Akinyele, Nigeria	811 (90)	59	7.3 (5.5–9.1)	4.6 (2.1–7.1)	90	6	[22]
Asia							
Qatar	3149 (97)	67	1.7 (1.7–1.8)	1.45 ^b^	51	6	[48]
Yazd, Iran	1990 (86)	87	4.4 (3.3–5.4)	1.6 (0.8–2.4)	90		[46]
Chinese, Singapore	1232 (72)	45	3.2 (2.3–4.1)^a^	1.1 (0.2–4.8)	62		[31]
Chinese, Singapore	3353 (73)	134	3.2 (2.7–3.9)^a^	0.7 ^b^	85	10	[38]
Malay, Singapore	3280 (79)	150	3.4 (3.3–3.5)^a^	2.2 ^b^	92	10	[36]
Indian, Singapore	3400 (76)	78	1.9 (1.5–2.5)^a^	1.3 ^b^	72	10	[35]
Beijing, China	4439 (83)	158	3.7 (3.1–4.2)	2.2 (1.5–3.0)	-	2	[37]
Kailu, China	5197 (87)	169	2.9 (2.0–3.8)^a^	2.0 (1.3–2.7)	66	7	[32]
Bhaktapur, Nepal	3991 (83)	75	1.8 (1.7–1.9)^a^	0.3 ^b^	96	2	[33]
Central India	4711 (80)	122	3.5 (2.8–4.1)	1.0 (0.5–1.6)	–	1	[34]
Australia							
Indigenous, Australia	1061 (64)	26	2.2 (1.6–3.6)	1.5 (0.4–2.5)	81	12	[47]
Europe							
Ponza, Italy	1034 (84)	39	3.8^b^	0 (0.0–1.7)	–		[42]
Egna-Neumarkt, Italy	4297 (74)	121	2.9^b^ approx	0.5 ^b^	–		[41]
Wroclaw, Poland	4853 (83)	79	1.6 (1.3–2.0)	0.4 (0.1–1.1)	71		[44]

*CI* confidence interval

^a^Adjusted rates

^b^95 % confidence interval not reported

– no data

The classification of glaucoma by pathophysiological mechanism based on angle morphology is important because POAG and PACG have different natural histories and different management strategies. In Nigeria POAG was the commonest type of glaucoma, as reported in other black populations [3, 15–22, 45].

It is acknowledged that communities in Nigeria, where prior diagnosis of glaucoma is low, have extremely little knowledge about glaucoma. Questions were, therefore, not asked on whether first-degree relatives had glaucoma as participants would be highly unlikely to know. In addition, the ISGEO classification does not take first-degree relatives into account.

It is noteworthy that at least half of the glaucoma eyes had an IOP less than the mean +2SD IOP (22 mmHg) of non-glaucoma participants. The important implication is that IOP is unable to differentiate between those with glaucoma and those without glaucoma.

A limitation of this study is that the ‘gold standard’ Humphrey field analyzer was not used as, unlike the portable FDT perimeter, it would not have been feasible to transport it to all examination centers especially in the terrain and environment of the survey. Nevertheless, we had an acceptable and reproducible test of visual function based on the central 20° field of vision. Another limitation was that pachymetry was not done. The data would have added more information on corneal thickness in relation to glaucoma. Interestingly, in the Barbados Eye Studies, corneal thickness tended to be thinner in the black participants than in the white participants but was not correlated to IOP [56]. However, as corneal thickness decreased, there was a higher likelihood of incident OAG [57]. Also, not all participants had dilated disc assessment or photographic disc grading as this was not possible given the large sample size of the study. The fundus camera produced non-stereoscopic images and monocular clues were used to determine optic disc and cup boundaries. Though this may have led to misclassification, most of the cases classified as glaucoma in this survey were “barn-door”. Even though there is a tendency for non-stereoscopic assessments to yield slightly varied optic disc parameters [58, 59], these differences were inconsistent and the agreement between stereoscopic and non-stereoscopic VCDR assessment were generally extremely good and repeatable [58]. A further limitation was that the fundus camera was not calibrated for disc size so VCDRs could not be adjusted for disc size. Technical difficulties in the field (faulty camera or generator) meant that disc images were not obtained in 616 participants when needed. High humidity damaged the mirror coating of gonioscopy lenses so that some eligible participants did not have gonioscopy performed and VH AC angle estimation was used instead. Hence, the proportions for angle-closure glaucoma and open-angle glaucoma were obtained only from participants that had gonioscopy. Additionally, lack of indentation gonioscopy, use of a one-mirror gonioscopy lens, and defining open-angle glaucoma as a visible Schwalbe’s line may have led to some misclassification of the type of glaucoma. The survey protocol indicated detailed eye examination of those with VCDR asymmetry of ≥0.2 whereas the asymmetry required for Level 1 diagnosis in later analysis was found to be 0.1. In individuals with VCDR asymmetry between 0.1 and 0.2, the diagnosis of glaucoma was based on the presence of glaucomatous visual fields. The ISGEO classification system is designed to identify moderate, severe glaucoma and those blind from glaucoma and therefore glaucoma ‘suspects’ and those with early disease may not have been captured. Our estimate is, therefore, a minimal estimate.

One survey team inadvertently used the C-20-1 FDT screening mode in 141 clusters. The C-20-1 mode has greater specificity and is less likely to misclassify a normal field. The C-20-5 has higher sensitivity at detecting early defects at the expense of lower specificity. To overcome the difference visual fields were classified according to the probability of pattern deviation and were equalized for the 2 screening modes.

Having described the high prevalence and distribution of glaucoma in this comprehensive and representative study, we are obliged to recommend a strategy for the prevention blindness and visual impairment from glaucoma in Nigeria and more widely in West Africa. The clinical care of glaucoma in Nigeria remains challenging and we suggest a top-down approach [60].

## Conclusion

This nationally representative survey in Nigeria indicates a high prevalence of glaucoma, with ethnic variation, severity at an earlier age and high rates of blindness. The latter is likely to reflect an aggressive natural history as well as lack of awareness of the condition and low levels of treatment. Most glaucoma in Nigeria is POAG with a high proportion of secondary glaucoma being the consequence of procedures for cataract. The findings shed light on the more severe and prevalent disease seen in black communities of the West African diaspora around the world and quantify the enormous challenge of preventing blindness from glaucoma in West Africa and in people of this ethnic origin. Public health control strategies with high quality integrated glaucoma care services will be required to reduce morbidity and blindness.

## Abbreviations

AC: anterior chamber
BIO: binocular indirect ophthalmoscopy
CI: confidence interval
FDT: frequency doubling technology
GPZ: geo-political zone
IOP: intraocular pressure
ISGEO: International Society of Geographical and Epidemiological Ophthalmology
MEHRC: Moorfields Eye Hospital Reading Centre
NC: North central
NE: North east
NW: North west
OAG: open-angle glaucoma
PACG: primary angle-closure glaucoma
PDP: pattern deviation plot
POAG: primary open-angle glaucoma
RAPD: relative afferent pupillary defect
SD: standard deviation
SE: South east
SS: South south
SW: South west
TDP: total deviation plot
VA: visual acuity
VCDR: vertical cup:disc ratio
VH: Van Herick’s
WHO: World Health Organization
